# Supplementary material for: Amine-responsive cellulose-based ratiometric fluorescent materials for real-time and visual detection of shrimp and crab freshness
Source: Nat Commun. 2019 Feb 15;10:795. doi: 10.1038/s41467-019-08675-3 (PMC6377604; doi:10.1038/s41467-019-08675-3)
Supplement: Supplementary file 2 — Supplementary information [file 41467_2019_8675_MOESM2_ESM.pdf]

Supplementary Information

Amine-responsive cellulose-based ratiometric fluorescent materials for real-time and visual detection of shrimp and crab freshness

Jia *et al.*

Supplementary Figures

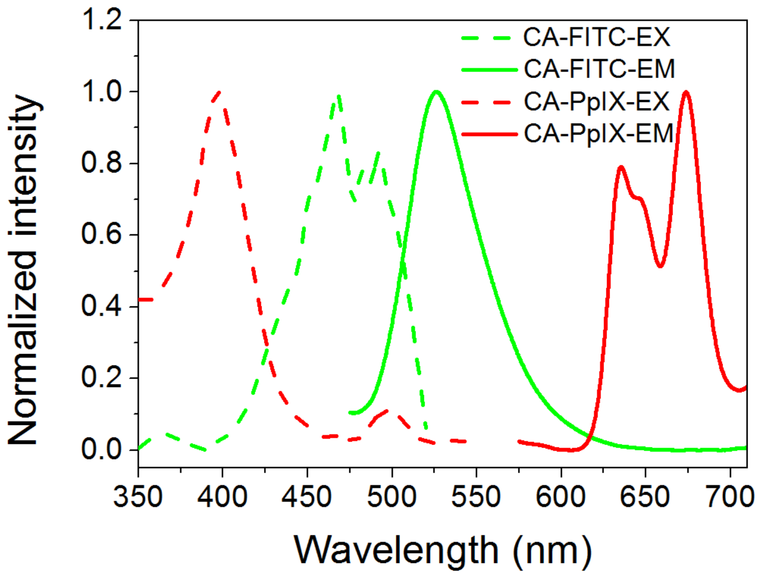

Supplementary Figure 1. Normalized excitation and emission spectra of CA-FITC and CA-PpIX.

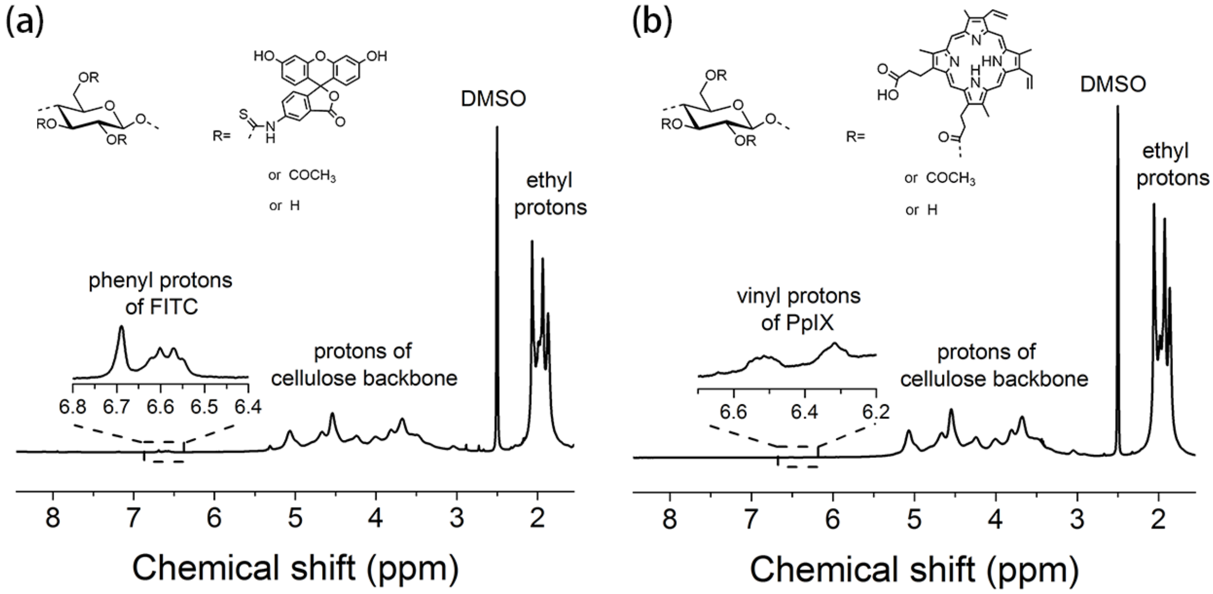

Supplementary Figure 2. ^{1}H-NMR spectra of (a) CA-FITC and (b) CA-PpIX.

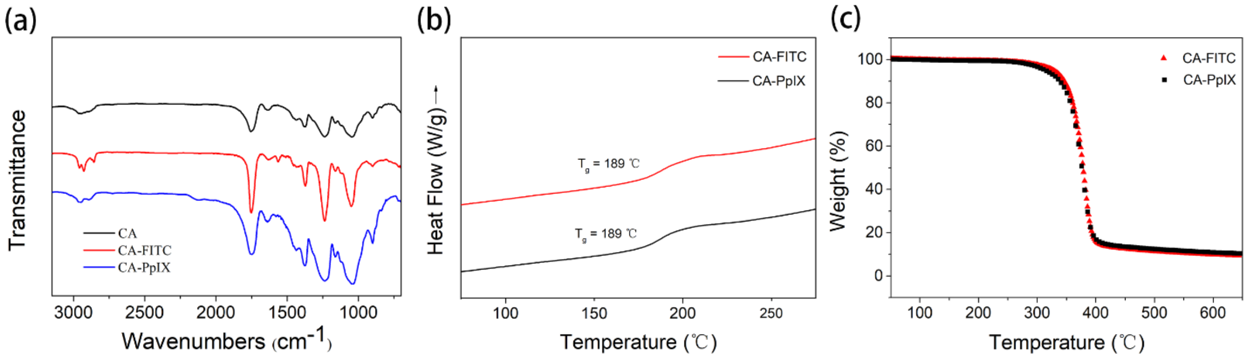

Supplementary Figure 3. Characterization of CA-FITC and CA-PpIX. (a) FTIR spectra of CA, CA-FITC and CA-PpIX. (b) DSC curves of CA-FITC and CA-PpIX. (c) TGA curves of CA-FITC and CA-PpIX.

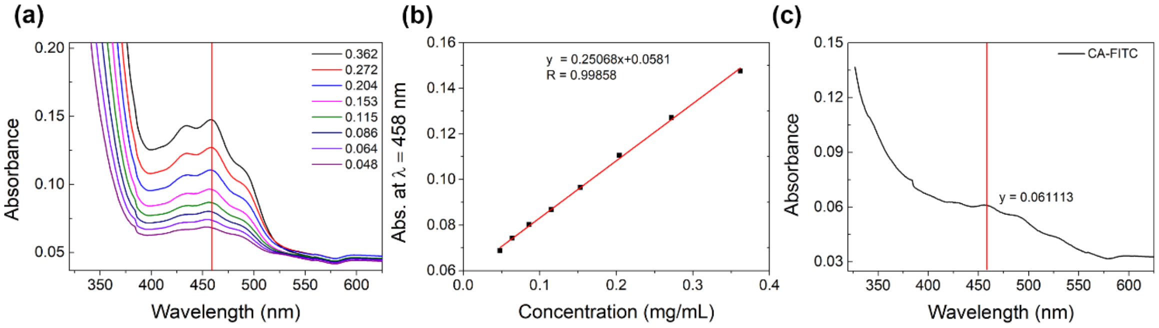

Supplementary Figure 4. Detection of the degree of substitution (DS) of CA-FITC. (a) UV-vis spectra of FITC in DMF with different concentrations (mg/mL). (b) Calibration curve of FITC concentration with respect to the absorbance at 458 nm. (c) UV-vis spectrum of CA-FITC.

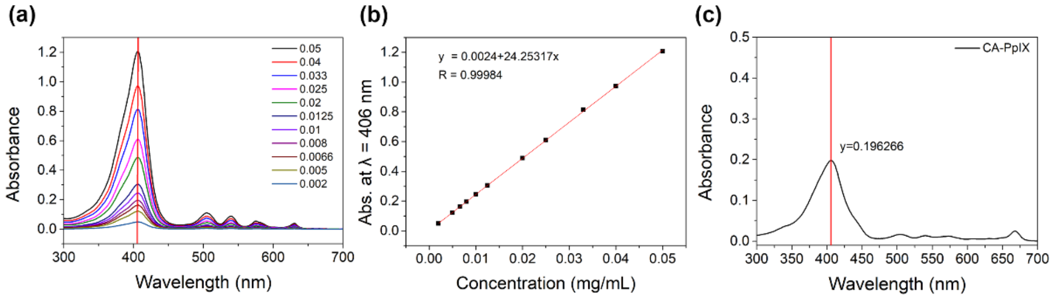

Supplementary Figure 5. Detection of the DS of CA-PpIX. (a) UV-vis spectra of PpIX in DMF with different concentrations (mg/mL). (b) Calibration curve of PpIX concentration with respect to the absorbance at 406 nm. (c) UV-vis spectrum of CA-PpIX.

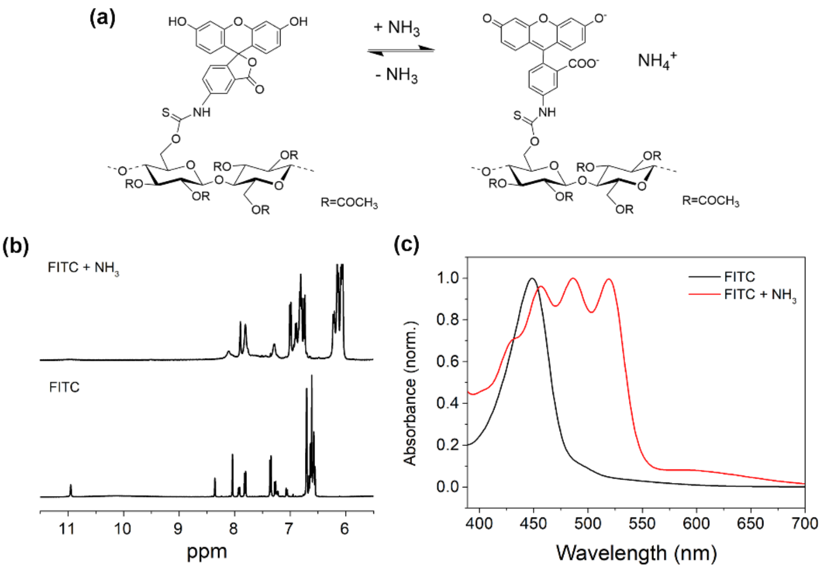

Supplementary Figure 6. Amine-response mechanism of CA-FITC. (a) Schematic illustration of the molecular structure change of CA-FITC upon reacting with NH_3. (b) ^{1}H-NMR spectra of FITC and FITC upon reacting with NH_3. (c) UV-vis spectrum of FITC and FITC upon reacting with NH_3.

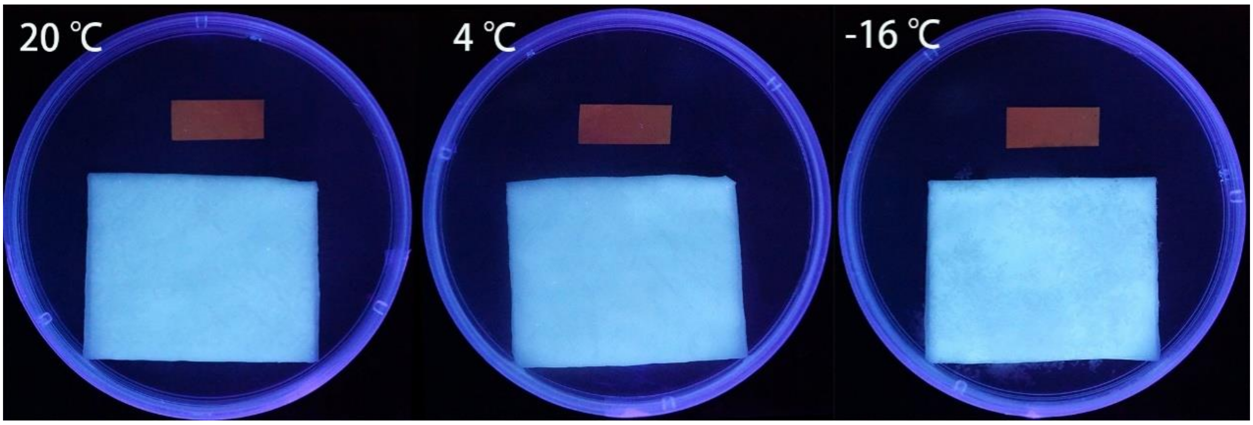

Supplementary Figure 7. Images of the electrospun membranes with wet tissues stored at different temperature for 5 days, and no fluorescence color change occurred.

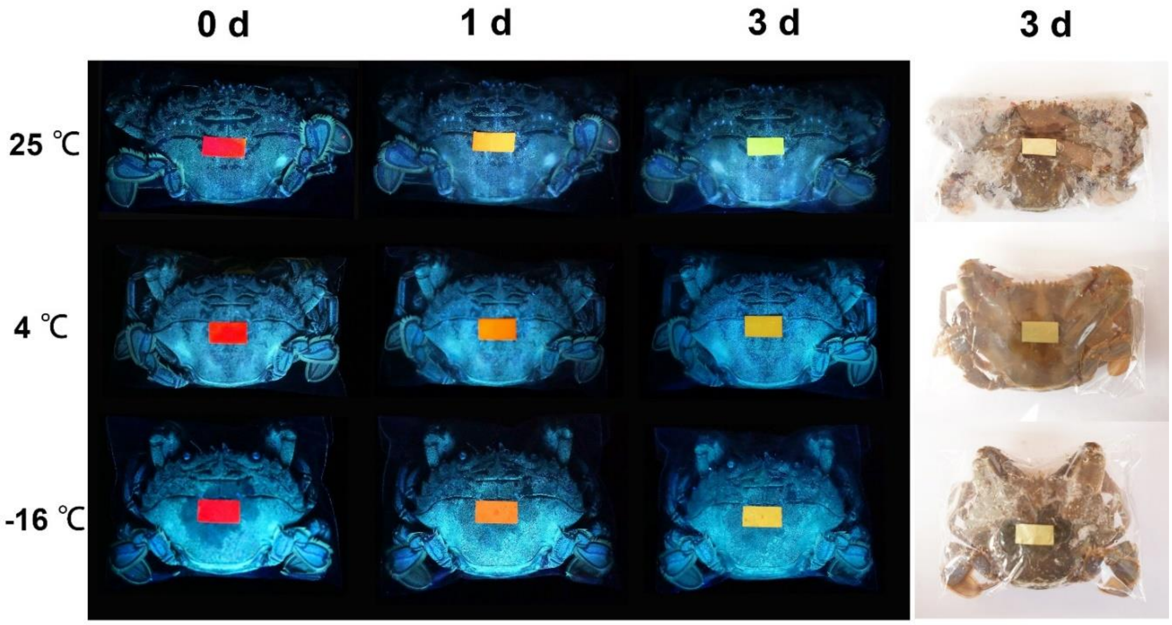

Supplementary Figure 8. Images of the cellulose-based ratiometric fluorescent material with a red initial fluorescence as a smart trademark for monitoring the freshness of crabs stored at different conditions.

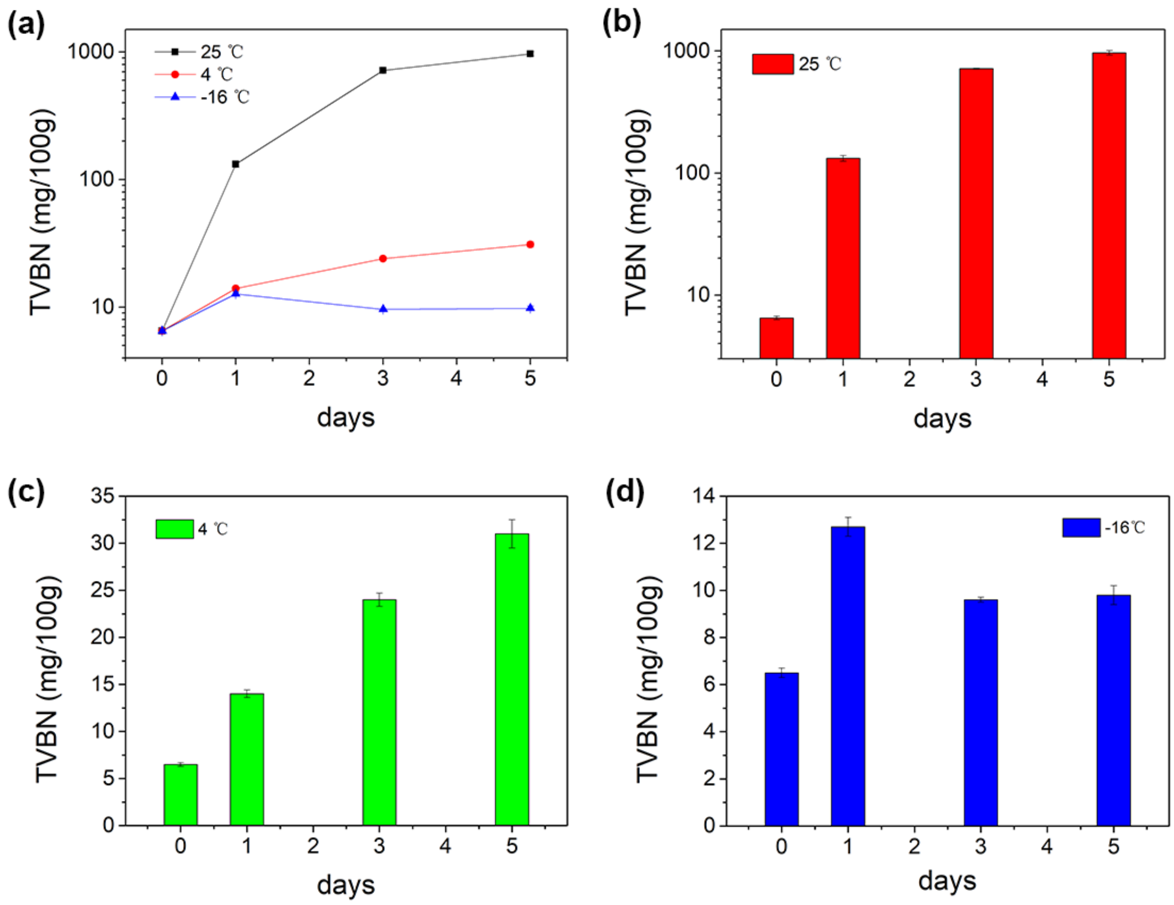

Supplementary Figure 9. (a) The total volatile basic nitrogen (TVBN) in shrimps stored at 25 °C, 4 °C and -16 °C for different days; (b-d) The TVBN content in shrimps stored at 25 °C, 4 °C and -16 °C respectively. Error bar represents the standard deviation, n = 3.

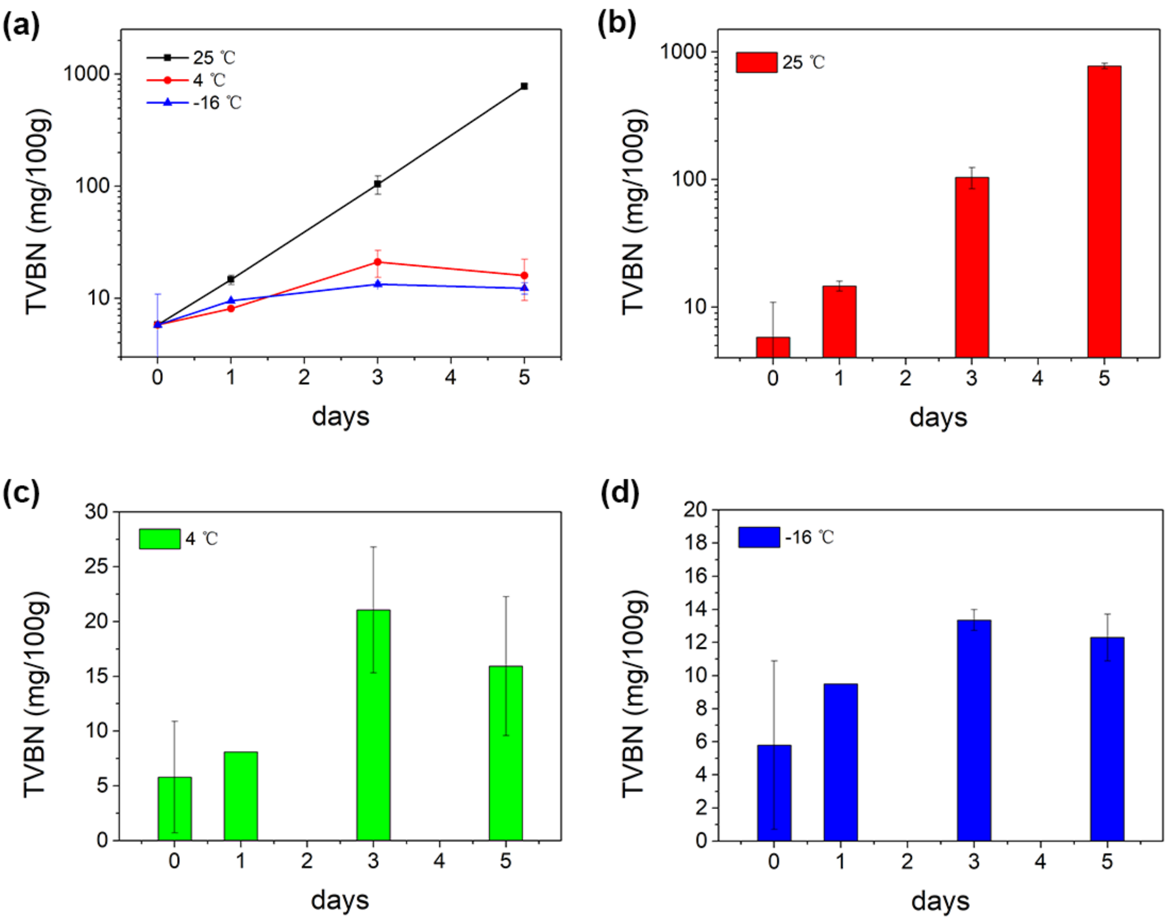

Supplementary Figure 10. (a) The total volatile basic nitrogen (TVBN) in crabs stored at 25 ℃, 4 ℃ and -16 ℃ for different days; (b-d) The TVBN content in crabs stored at 25 ℃, 4 ℃ and -16 ℃ respectively. Error bar represents the standard deviation, n = 3.

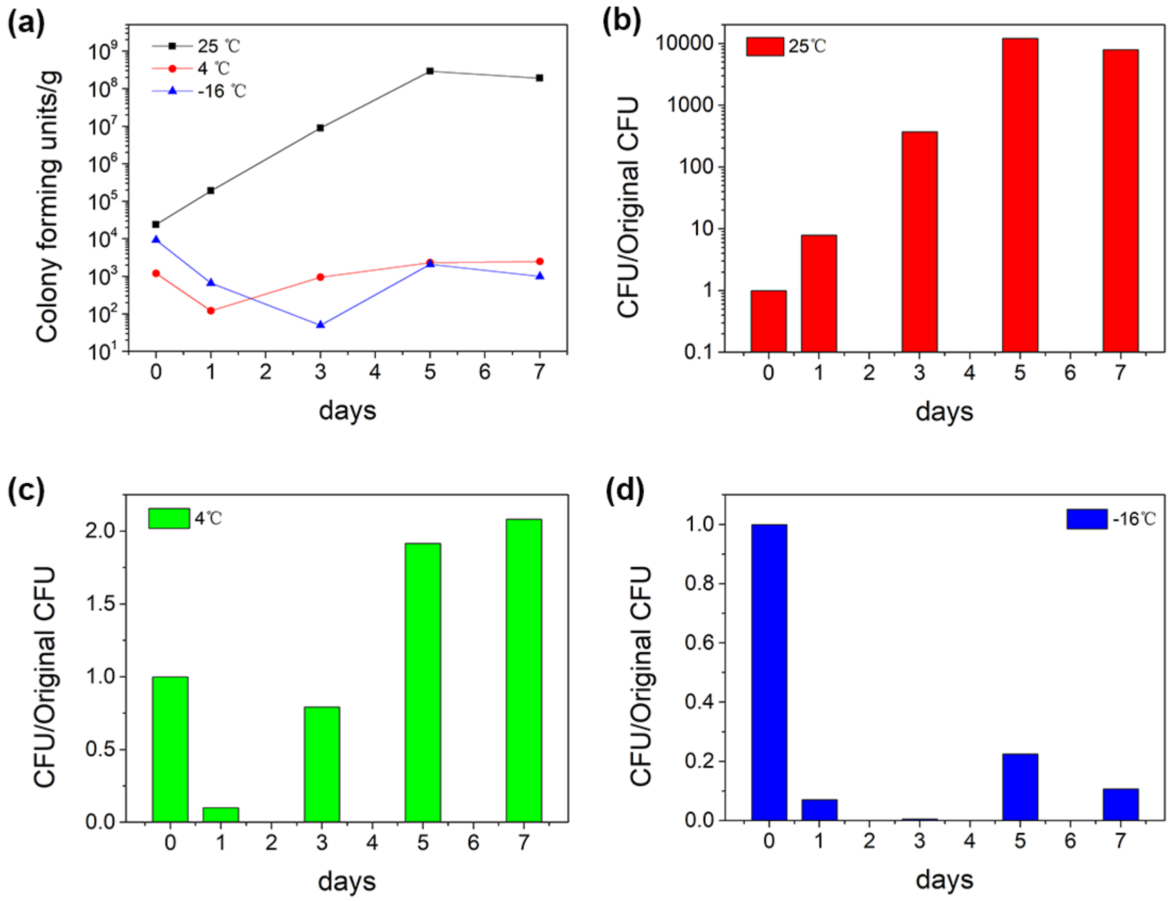

Supplementary Figure 11. (a) Colony forming units (CFU) of bacteria in shrimps stored at 25 °C, 4 °C and -16 °C for different days; (b-d) The ratio of CFU and original CFU (fresh shrimps, 0 days) of bacteria in shrimps stored at 25 °C, 4 °C and -16 °C for different days.

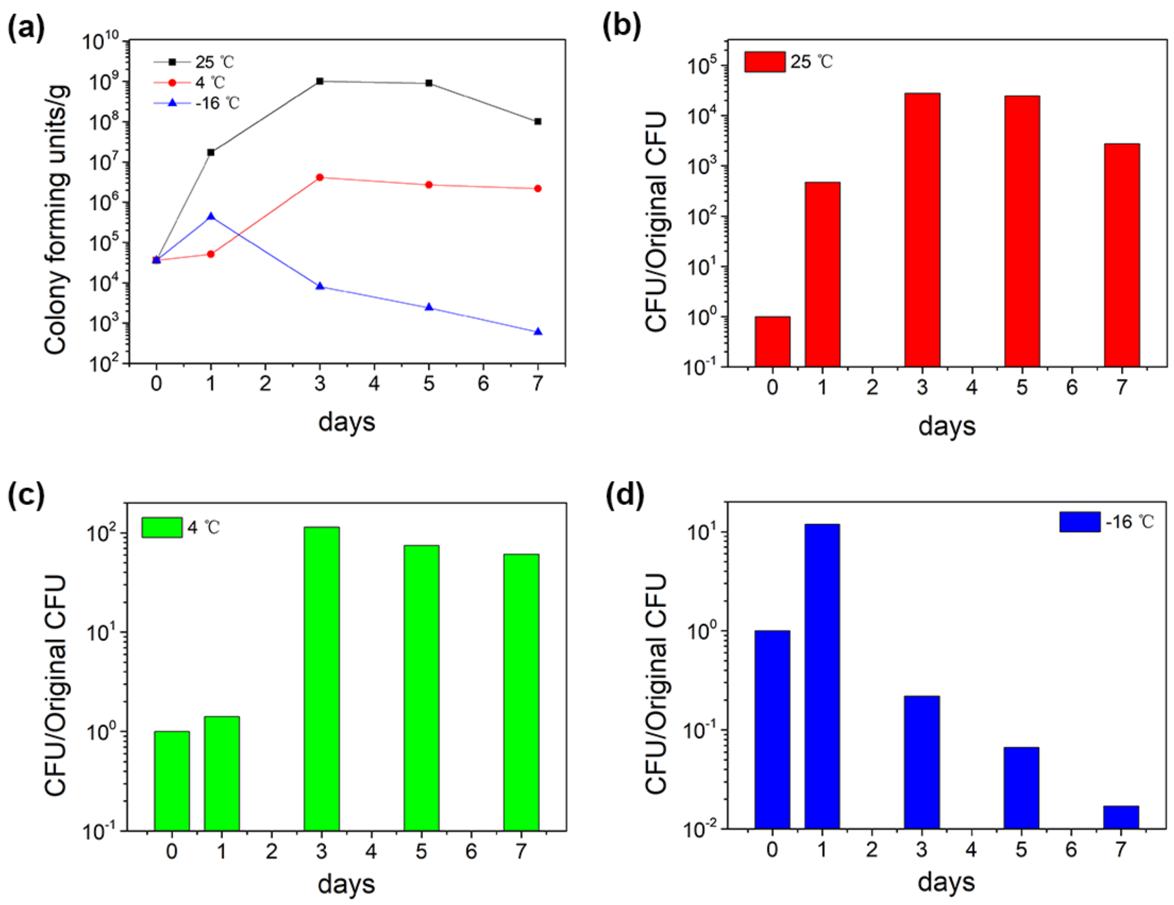

Supplementary Figure 12. (a) Colony forming units (CFU) of bacteria in crabs stored at 25 °C, 4 °C and -16 °C for different days; (b-d) The ratio of CFU and original CFU (fresh crabs, 0 days) of bacteria in crabs stored at 25 °C, 4 °C and -16 °C for different days.
