# Supplementary material for: Amine-responsive cellulose-based ratiometric fluorescent materials for real-time and visual detection of shrimp and crab freshness
Source: Nat Commun. 2019 Feb 15;10:795. doi: 10.1038/s41467-019-08675-3 (PMC6377604; doi:10.1038/s41467-019-08675-3)
Supplement: Supplementary file 3 — Description of Additional Supplementary Files [file 41467_2019_8675_MOESM3_ESM.pdf]

Description of Additional Supplementary Files

File Name: Supplementary Movie 1
Description: The response behavior of fluorescent Cellulose materials.
